# Resting-state EEG delta and alpha power predict response to cognitive behavioral therapy in depression: a Canadian biomarker integration network for depression study

**DOI:** 10.1038/s41598-023-35179-4

**Published:** 2023-05-24

**Authors:** Benjamin Schwartzmann, Lena C. Quilty, Prabhjot Dhami, Rudolf Uher, Timothy A. Allen, Stefan Kloiber, Raymond W. Lam, Benicio N. Frey, Roumen Milev, Daniel J. Müller, Claudio N. Soares, Jane A. Foster, Susan Rotzinger, Sidney H. Kennedy, Faranak Farzan

**Affiliations:** 1 eBrain Lab, School of Mechatronic Systems Engineering, Simon Fraser University, 13750-96 Ave, Surrey, BC V3V 1Z2 Canada; 2 University of Toronto, 27 King’s College Circle, Toronto, ON M5S 1A1 Canada; 3 Centre for Addiction and Mental Health, 1001 Queen St. W, Toronto, ON M6J 1H4 Canada; 4 Department of Psychiatry, Dalhousie University, 5909 Veterans’ Memorial Lane, Halifax, NS B3H 2E2 Canada; 5 Department of Psychiatry, University of British Columbia, 2255 Wesbrook Mall, Vancouver, BC V6T 2A1 Canada; 6 Department of Psychiatry and Behavioural Neurosciences, McMaster University, 100 West 5th St., Hamilton, ON L8N 3K7 Canada; 7 Mood Disorders Program and Women’s Health Concerns Clinic, St. Joseph’s Healthcare, 100 West 5th St., Hamilton, ON L8N 3K7 Canada; 8 Department of Psychiatry, Providence Care, Queen’s University, 752 King Street West, Kingston, ON K7L 4X3 Canada; 9 Unity Health Toronto, Toronto, ON Canada; 10 University Health Network, 399 Bathurst Street, Toronto, ON M5T 2S8 Canada

**Keywords:** Depression, Predictive markers, Neural circuits

## Abstract

Cognitive behavioral therapy (CBT) is often recommended as a first-line treatment in depression. However, access to CBT remains limited, and up to 50% of patients do not benefit from this therapy. Identifying biomarkers that can predict which patients will respond to CBT may assist in designing optimal treatment allocation strategies. In a Canadian Biomarker Integration Network for Depression (CAN-BIND) study, forty-one adults with depression were recruited to undergo a 16-week course of CBT with thirty having resting-state electroencephalography (EEG) recorded at baseline and week 2 of therapy. Successful clinical response to CBT was defined as a 50% or greater reduction in Montgomery-Åsberg Depression Rating Scale (MADRS) score from baseline to post-treatment completion. EEG relative power spectral measures were analyzed at baseline, week 2, and as early changes from baseline to week 2. At baseline, lower relative delta (0.5–4 Hz) power was observed in responders. This difference was predictive of successful clinical response to CBT. Furthermore, responders exhibited an early increase in relative delta power and a decrease in relative alpha (8–12 Hz) power compared to non-responders. These changes were also found to be good predictors of response to the therapy. These findings showed the potential utility of resting-state EEG in predicting CBT outcomes. They also further reinforce the promise of an EEG-based clinical decision-making tool to support treatment decisions for each patient.

## Introduction

Major depressive disorder (MDD) affects more than 300 million people worldwide^1,2^ and is projected to become the leading cause of disability by 2030^3^. Among the variety of pharmacological and psychotherapeutic treatments available, cognitive behavioral therapy (CBT) is often recommended as a first-line treatment for MDD^4–6^. This well-established intervention teaches patients to identify and modify thoughts and behaviors to reduce maladaptive patterns and enhance mood and well-being^7^. CBT is an effective treatment option^8–10^ with enduring effects^11^. Nevertheless, while CBT is comparable in effectiveness to pharmacotherapy, up to 50% of patients with MDD fail to achieve remission following CBT or achieve only partial response^12^. Due to the heterogeneity of depression, there is no current method to determine if CBT is likely to benefit a given individual. As a result, some patients may end up spending months in a therapy that will not alleviate their depressive symptoms. Moreover, access to publicly-funded CBT is limited in many countries, and certified cognitive-behavioral therapists are scarce, hampering access to this therapy^13–17^. To reduce the time spent in ineffective therapy and optimize resource allocation within health systems, it is essential to identify which patients are likely to benefit from this therapy.

Recently, electroencephalography (EEG) was used to identify biomarkers that can predict the response to pharmacological and brain stimulation treatments^18–23^. In this regard, previous research has investigated the predictive utility of several resting-state EEG measures, such as band power, cordance (a measure of band power normalized in both spatial and frequency dimensions) or asymmetry (a measure derived from calculating the ratio between band power in left and right hemispheres).

Early studies identified an association between lower baseline delta power and response to tricyclic and selective serotonin reuptake inhibitor (SSRI) antidepressants, imipramine and paroxetine, respectively^24,25^. In contrast, a recent study found greater baseline delta oscillations to be predictive of improvement in loss of insight following treatment with several SSRIs and the serotonin-norepinephrine reuptake inhibitor (SNRI) venlafaxine^23^. An increase in delta activity after a few weeks of SSRI treatments is likewise associated with treatment response^21,26^. An increase in slow oscillation (including delta) was also observed following electroconvulsive therapy (ECT) but results were not specific to responders to the treatment^20,27,28^.

The link between theta activity and response to antidepressants is mixed. Baseline theta power was lower in responders to a tricyclic (imipramine), several SSRIs, and an SNRI (venlafaxine)^24,29^. On the other hand, greater baseline theta power was found in responders to another tricyclic (nortriptyline), several SSRIs, and rTMS^22,30–33^. Responders to the SSRI paroxetine, and combined treatment with the SSRI escitalopram and bupropion (a norepinephrine/dopamine-reuptake inhibitor (NRI)) exhibited an early increase in theta power^26,34^. However, early reduction in theta cordance was also observed in responders to venlafaxine, fluoxetine (SSRI), and bupropion^35–41^. Theta cordance has also been shown to both reduce and increase in responders to rTMS after 1 week of treatment^37,42^. These inconsistencies across studies could be explained by distinct treatment mechanisms but could also be due to differences in age ranges, and differences in rTMS treatment protocols (10 Hz left-dorsolateral prefrontal cortex rTMS in the Bares study, versus 1 Hz right-dorsolateral prefrontal cortex rTMS in the Hunter study).

A number of authors have reported an association between increased baseline alpha oscillations and response to different antidepressant medication types (including fluoxetine, tricyclics imipramine and amitriptyline, and combined treatment with escitalopram and bupropion) in addition to rTMS treatment^24,34,43–47^. A reduction in alpha power after several weeks of treatment has also been reported in responders to several SSRIs^21,26,34^. Similarly, a decrease in activity at high frequencies (including alpha) was observed following ECT treatment^20,28^. Some studies have also probed the predictive utility of alpha asymmetry to antidepressant treatments, although these results are inconsistent^21,34,43–46,48^. One study also investigated alpha asymmetry as a potential biomarker of response to CBT, but this was done in the context of anxiety, and not depression^49^. It is also interesting to note that some studies found evidence of greater left hemisphere advantage for verbal dichotic listening in responders than non-responders to CBT^50–52^. However, these studies did not used EEG measures and it is unclear if the findings are related specifically to asymmetry in the alpha band.

Finally, findings in other frequency bands have been scarce: a trend for greater beta power in responders to imipramine and paroxetine was reported in early studies^24,25^; and an increase in gamma connectivity after 1 week of treatment was observed in responders to rTMS^33^.

Despite some promising results, so far as we are aware no study to date has investigated the predictive utility of resting EEG measures prior to CBT for depression. Identifying biomarkers that can help predict the response to this widely used psychotherapy may assist in the development of more effective personalized treatments. Based on previous results, greater alpha power at baseline and reduction in the alpha activity in responders appear to be a common finding across other treatment modalities and could be a general marker of response to antidepressant treatment, including CBT. However, findings in other frequency bands could be more treatment specific. In this regard, one mechanism specific to CBT may involve changes in cognitive control^53–57^. For example, an alteration in neural correlates of inhibitory control following therapy has been reported in a recent study^56^. Considering that low-frequency oscillations have been associated with cognitive control in previous literature^58–67^, delta and theta activity could also be expected to be markers of response to CBT.

Here, we sought to assess whether spectral features derived from resting-state EEG could be used to classify responders and non-responders to CBT. In a study by the Canadian Biomarker Integration Network for Depression (CAN-BIND), adults with MDD were recruited to undergo 16 weeks of CBT. Resting-state EEG signals were recorded for each participant at baseline (week 0), week 2 of therapy, and following completion of the therapy course (week 16). Here, EEG data at baseline was used to identify potential biomarkers of response to CBT. We also used data at week 2 to investigate whether early changes in EEG oscillatory activity would help predicting response to the therapy. A participant was categorized as a responder if the reduction in their Montgomery-Åsberg Depression Rating Scale (MADRS) scores from baseline to post-treatment therapy was equal to or more than 50%, and as a non-responder, if the reduction was less than 50%. We hypothesized that power spectral EEG measures would differ between subsequent responders and non-responders in exhibiting greater alpha power at baseline and an early reduction in alpha activity. We also speculated that response to CBT would be associated with lower delta and theta power at baseline and an early increase in delta and theta activity.

## Results

### Demographic and clinical characteristics

In total, forty-one participants were recruited for the study. Thirty-seven completed clinical assessments at baseline and at the end of the therapy. Thirty participants completed EEG sessions at both baseline and week 2 (three participants missed the EEG session at baseline, and four participants missed the EEG session at week 2) and were included in the analysis (Table 1).

**Table 1:** Demographics and clinical characteristics of responders and non-responders.

Variable	Total (n = 30)		Responders (n = 16)		Non-responders (n = 14)		Test of difference	
	n	(%)	n	(%)	n	(%)	X^2^	P
Female	23	76.7	14	87.5	9	64.3	2.249	0.134
White/European	18	60.0	13	81.2	5	35.7	6.451	0.011

	Mean	SD	Mean	SD	Mean	SD	t	P
Age	39.1	10.6	40.6	12.6	37.3	7.9	0.855	0.400
MADRS week 0	30.3	8.4	27.3	6.1	28.7	10.3	− 0.471	0.641
MADRS week 2	33.8	7.4	26.3	6.6	27.6	8.4	− 0.509	0.615
MADRS week 16	15.1	9.5	6.8	4.3	21.4	7.5	− 6.717	< 0.001
% Change in MADRS	48.4	33.3	75.8	13.5	21.9	24.4	7.606	< 0.001

Of the thirty participants considered in this study, sixteen (53%) were responders, and fourteen (47%) were non-responders. Groups did not differ by sex (p = 0.134) or age (p = 0.400). However, responders and non-responders differed by ethnicity (p = 0.011). No differences were observed between groups in MADRS scores at baseline and week 2 (p = 0.641 and p = 0.615, respectively).

### Relative power EEG spectra

The average relative power EEG spectra for responders and non-responders at baseline and week 2 can be found in Supplementary Fig S1. Relative power measures showed excellent internal consistency with an averaged Cronbach’s alpha across frequencies and electrodes of 0.911 at baseline, and 0.925 at week 2, respectively (Supplementary Fig S2).

### Differences in relative power

Analysis of variance showed a significant (p < 0.05) interaction effect of CBT Response x Time [mean F(1, 28) = 5.747 (4.197 to 17.358)] across several frequencies and channels in the sensor space. A significant interaction effect of CBT Response x Time [mean F(1, 28) = 5.791 (4.201 to 14.159)] was also observed across several frequencies and regions of interest in the source space. As such, several *post-hoc* analyses were conducted. In what follows, the results of *post-hoc* *t* tests, correlational and predictive analyses are presented for baseline, week 2 and change in baseline to week 2. For each analysis, cluster-based permutation testing was applied to correct for multiple comparisons.

### Relative power at baseline

**Figure 1:**
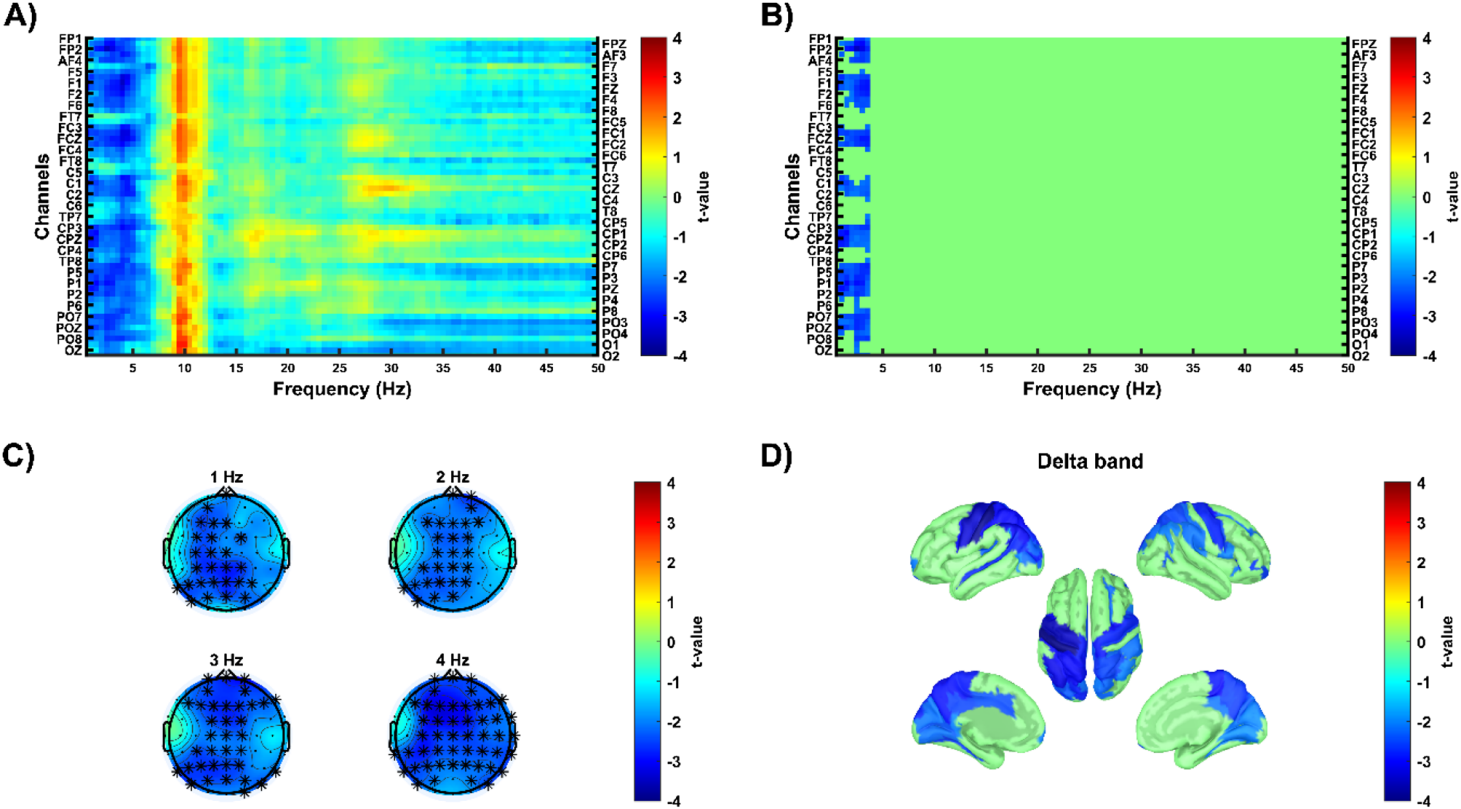
Differences in relative power at baseline between responders and non-responders. Cold colors show lower relative power in responders compared to non-responders. Warm colours show higher relative power in responders compared to non-responders. (**A,B**) The x-axis shows frequencies from 0.5 to 50 Hz. The y-axis shows all electrodes from 1 to 58. Image A shows uncorrected t-value map, image B shows significant clusters (p < 0.025, single-tailed, cluster corrected for multiple comparisons) (**C**) Topographies illustrate t-values at different frequencies with stars indicating electrodes that belonged to the significant cluster. (**D**) Cortical maps depict source-localized regions (p < 0.05, uncorrected) in the frequency band in which the cluster was found at the sensor space level.

At baseline, *post-hoc* analysis revealed a group difference in delta band, with responders exhibiting lower delta activity compared to non-responders (negative cluster, p = 0.016, Cohen d = 0.882, Fig. 1). In the source space, this difference was identified in several brain areas, including bilateral precentral gyrus and sulcus, bilateral central sulcus, bilateral postcentral gyrus and sulcus, bilateral precuneus, bilateral superior and inferior parietal gyri, bilateral middle occipital gyri, left midcingulate cortex (MCC), and bilateral posterior cingulate cortex (PCC) (Fig. 1).

**Figure 2:**
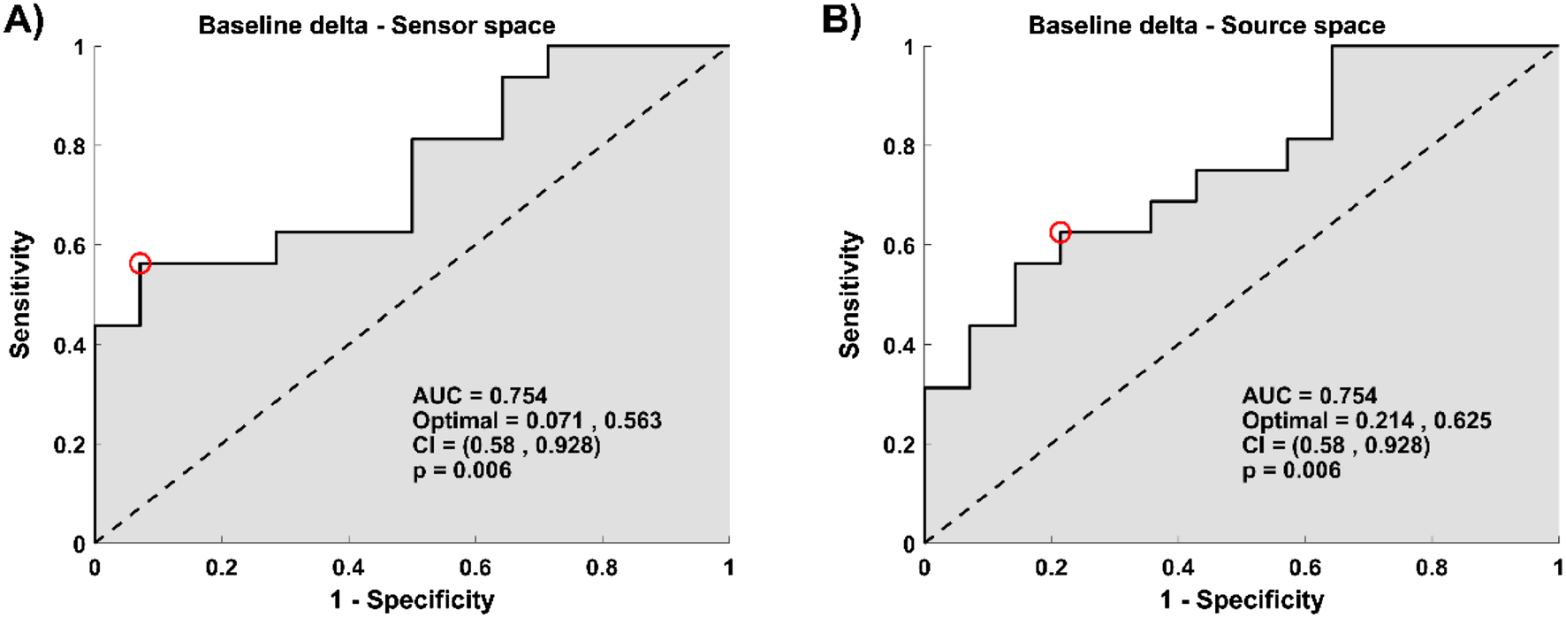
Relative power in the delta band at baseline predicts improvement in depressive symptoms. (**A**) The plot depicts the ROC curve across all possible threshold values of the predictor for average power relative value within the cluster found in the delta band at the sensor space (**B**) The plot depicts the ROC curve across all possible threshold values of the predictor for average power relative value in the delta band across regions found significant at the source space. (**A**,**B**) In both plots, x-axes represent false-positive rates (1-specificity), y-axes the true positive values (sensitivity). Here, specificity corresponds to percentage of non-responders who were predicted to be non-responders, and sensitivity corresponds to percentage of responders who were predicted to be responders. The red circle shows the optimum operating point of the ROC curve.

The difference observed between groups in the delta band at baseline was found to be predictive of the treatment outcome (AUC = 0.754, p = 0.006 in the sensor space, and AUC = 0.754, p = 0.006 in the source space, Fig. 2).

Results were similar when including all participants who completed the EEG session at baseline (thirty-four participants) and can be found in Supplementary Figs S3 and S4.

### Relative power at week 2

At week 2, *post-hoc* analysis did not reveal any differences between groups in relative power. There was also no significant correlation between relative power at week 2 and improvement in depressive symptoms.

Results did not differ when including all thirty-three participants who completed the EEG session at week 2 (thirty-three participants).

### Early changes in relative power

**Figure 3:**
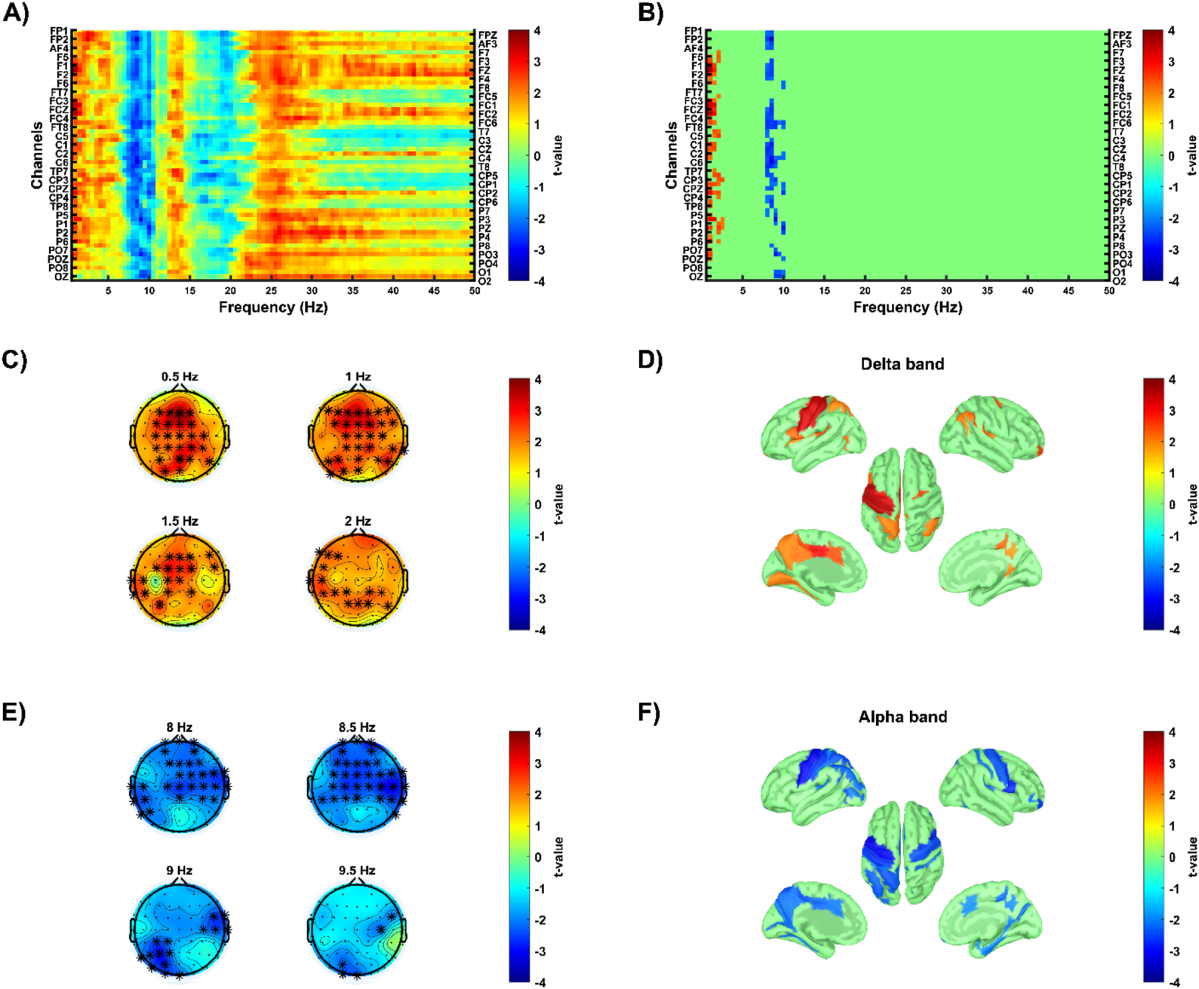
Differences in relative power early changes between responders and non-responders. Cold colors show lower relative power changes in responders compared to non-responders. Warm colours show higher relative power changes in responders compared to non-responders. (**A**,**B**) The x-axis shows frequencies from 0.5 to 50 Hz. The y-axis shows all electrodes from 1 to 58. Image A shows uncorrected t-value map, image B shows significant clusters (p < 0.025, single-tailed) using cluster-based correction. (**C**,**E**) Topographies illustrate t-values at different frequencies with stars indicating electrodes that belonged to the significant cluster. (**D**,**F**) Cortical maps depict source-localized regions (p < 0.05) in the frequency band in which the cluster was found at the sensor space level.

*Post-hoc* analysis revealed group differences in delta and alpha bands, with responders exhibiting an increase in delta activity (positive cluster, p = 0.020, Cohen d = 1.118, Fig. 3) and a decrease in alpha activity (negative cluster, p = 0.014, Cohen d = 0.949, Fig. 3) relative to non-responders. Both differences were localized to similar brain regions, including left precentral, postcentral, and superior parietal gyri, left central sulcus, left precuneus, and left MCC and PCC. In addition, decrease in alpha was also localized to right precentral gyrus and left inferior parietal gyrus (Fig. 3).

**Figure 4:**
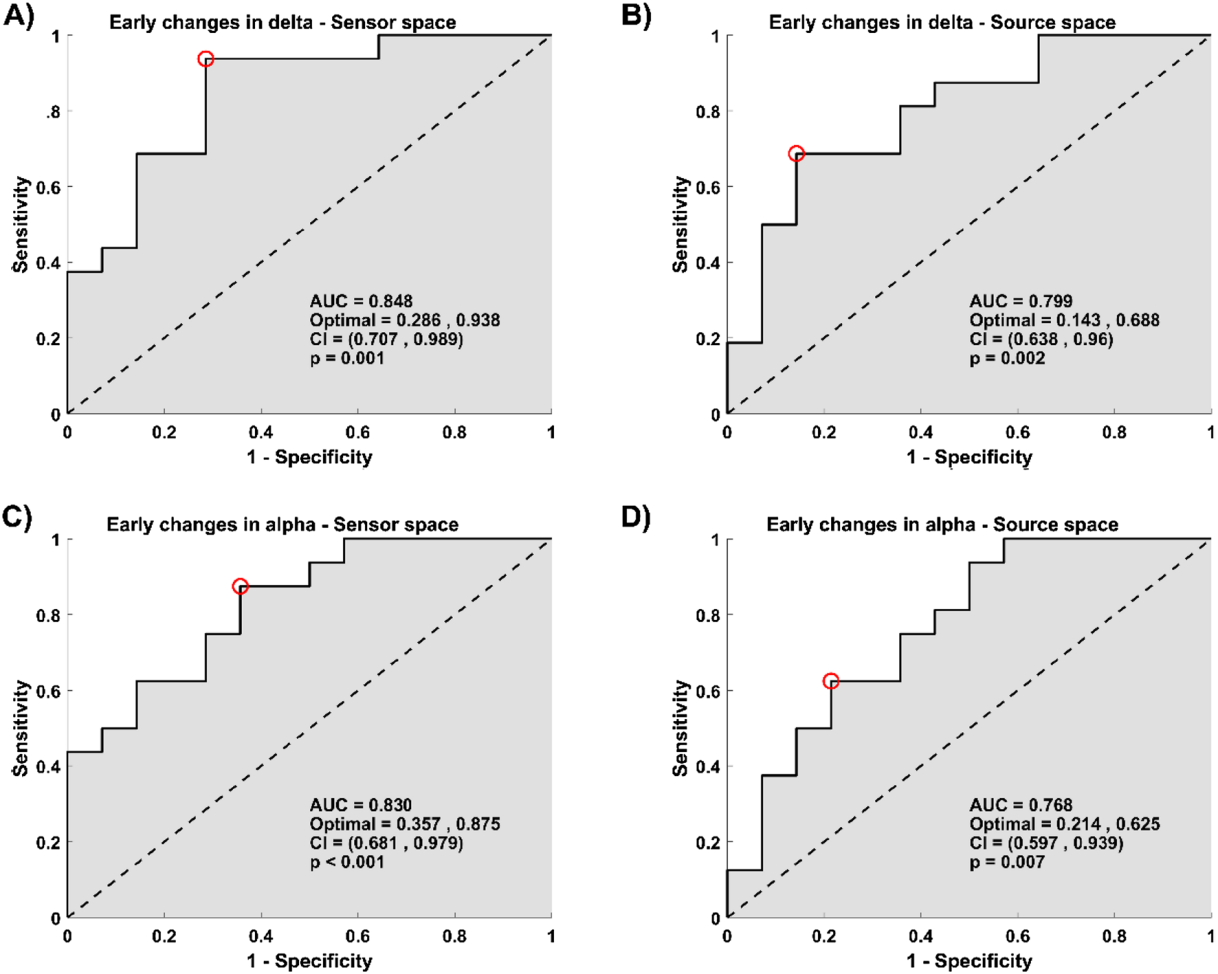
Early changes in the delta and alpha relative power bands predict improvement in depressive symptoms. **(A**,**C**) The plots depict the ROC curve across all possible threshold values of the predictor for average power relative value within the clusters found in the delta band (**A**), and in the alpha band (**C**) at the sensor space. (**B**,**D**) The plots depict the ROC curve across all possible threshold values of the predictor for average power relative value in the delta band (**B**) and the alpha band (**D**) across regions found significant at the source space. (**A**–**D**) In all plots, x-axes represent false positive rates (1-specificity), y-axes the true positive values (sensitivity). Here, specificity corresponds to the percentage of non-responders who were predicted to be non-responders, and sensitivity corresponds to the percentage of responders who were predicted to be responders. The red circle shows the optimum operating point of the ROC curve.

Early changes observed in delta and alpha activity were good predictors of response to CBT. The significant prediction models had the following characteristic: early changes in delta activity model (AUC = 0.848, p = 0.001 in the sensor space, and AUC = 0.799, p = 0.002 in the source space, Fig. 4), and early changes in the alpha activity model (AUC = 0.830, p < 0.001 in the sensor space, AUC = 0.768, p = 0.007 in the source space, Fig. 4).

## Discussion

In this study, we showed that resting-state EEG delta and alpha power might have possibly moderate to strong predictive utility in predicting response to CBT for adults with depression.

A reduced baseline delta power was observed in responders and was predictive of response to CBT. Responders also exhibited an early increase in delta power, which also proved to be a good predictor of response to the therapy. While previous authors have also reported an association between slow oscillations and response to other antidepressant treatment modalities^20,21,23–28^, results are not unidirectional and may be related to different effects and mechanisms of antidepressants and different subtypes within depression. Here, the findings in the delta activity might reflect cognitive control changes, which have been attributed to the mechanisms of CBT^53–57^. Previous studies have shown the functional significance of delta activity during cognitive processing^59–67^. For example, delta oscillations may play a role in motivational and emotional processes^61,66^ and also in inhibitory control and response inhibition^59,60,63,65,67^. An increase in delta activity may also be an indicator of attention to internal processing during the performance of a mental task^62^. Regarding delta activity at rest, some previous studies reported the relevance of low-frequency intrinsic activity in the neural mechanisms of cognitive control processes^68–70^. In these studies, resting-state delta power was shown to be associated with behavioral performance and neural correlates of response inhibition during a go/no-go task. In another recent study, resting-state delta oscillations were predictive of good performance on an attentional set-shifting task^71^. The results here could suggest that CBT enhances cognitive control manipulation processes in patients who display an increase in delta activity after 2 weeks of therapy. Moreover, some studies involving meditation also reported an increase in delta activity during the suppression of interference^72,73^. In these studies, stronger delta activity in the medial prefrontal cortex, during the eyes-closed resting state, was suggested to reflect a reduction of emotional and cognitive engagement. The findings here could suggest that patients with stronger emotional engagement at baseline represent a subgroup who responds well to CBT. This correlates with a study in which patients presenting excessive attention toward aversive information were more likely to respond to CBT^74^. Increased attention to aversive stimuli is suggested to be a mechanism underlying emotional disorders in patients with depression^75^. The early increase in delta observed here in responders to CBT could therefore reflect a mechanism whereby CBT reduces negative emotional engagement that interferes with decision making and situational goals. Furthermore, when investigating if differences observed in early changes were associated with improvements in specific symptoms using subdomains of MADRS, early increases in delta were found to be related to improvement in sadness, and negative thoughts subdomains (Supplementary Fig S5). In comparison, early decreases in alpha were not related to any subdomains. While further analyses are necessary to better understand the role of delta oscillations, these results support the potential link between delta activity and cognitive control when processing negative information.

A significant difference was also found in the early changes in relative alpha power, with a decrease observed in responders. This change was found to have strong utility in predicting response to CBT. Reduction in alpha power has been shown to be a mechanistic marker of response to various modalities of antidepressant treatments (including medications, rTMS and ECT) across studies^20,21,28,34,47^. There is an inverse relationship between alpha power and cortical activity^76,77^. Hence, a decrease in alpha power could reflect an increase in cortical arousal toward the processing of external stimuli and cognitive engagement instead of an internally self-focused emotional processing^78–81^. Moreover, as this change was primarily over regions in left hemisphere, it could also indicate that cognitive therapies, which are highly verbal treatments, may involve cognitive processes meditated by regions in left hemisphere. This is in line with studies that found a left hemisphere advantage for verbal processing in responders to CBT^50–52^.

Altogether, it is possible that CBT may be better suited for individuals with overactive cognitive presentations. In this study, responders to the therapy were found to be more likely to exhibit lower delta activity and higher alpha activity, which could indicate greater inward-focused and self-referential processing. By using CBT to alter thought patterns, it might be possible to induce changes in brain activity, leading to improved mood and emotional states.

This study has certain limitations worth noting. First, considering the small sample size, any generalization from the findings should be made with caution. It also precludes the analysis of gender-based effects or other potential moderators such as ethnicity or clinical features. Secondly, placebo effects are unknown as findings were obtained during an open-label treatment. Replication of the results in a larger sample size and with a treatment control group is required. It would also inform the quantification of the differences in EEG predictors across treatment modalities.

In summary, this is the first study to show findings possibly indicated a potential utility of resting EEG in predicting response to CBT for adults with depression. The findings suggest that baseline measures and early changes in delta and alpha frequency bands might be used to classify responders and non-responders to CBT. While baseline measures might not be enough to predict CBT treatment outcomes accurately, information that arises during the early course of therapy was found to be a strong predictor of response to CBT. Given the limited access to in-person CBT therapy and the limited number of CBT therapists, being able to make an informative decision after the two first weeks of therapy about whether to continue or switch to another treatment would have great clinical value. EEG technology is becoming more accessible with more portable and less expensive devices^82,83^, and research investigating EEG-based biomarkers can inform its strategic application in clinical contexts. Future studies are required in order to validate the results, but the insight gained here may help the development of objective clinical decision tools to guide individual patients with MDD to the optimal treatment.

## Methods

### Participant sample

#### Recruitment

Participants were recruited at the Centre for Addiction and Mental Health (CAMH) using a research registry, internal referrals, and waitlists for the CAMH Mood and Anxiety Program. All research was conducted in accordance with the Declaration of Helsinki, and all participants gave informed written consent prior to enrolment in the study. The protocol was approved by the Centre for Addiction and Mental Health Research Ethics Board and was registered with clinicaltrials.gov (https://clinicaltrials.gov/ct2/show/NCT02883257).

#### Eligibility for CBT

Participants had to be 18 years or older to be enrolled in the study. Inclusion criteria included: (1) clinical diagnosis of MDD or Persistent Depressive Disorder according to the DSM-5; (2) fluency in English; (3) capacity to give informed consent. The exclusion criteria can be found in the Supplementary Information.

#### CBT

CBT was provided on an individual basis by a registered psychologist or graduate-level trainee under the direct supervision of a registered psychologist. Participants received 20 sessions over approximately 16 weeks, with 2 sessions per week in the first 4 weeks and 1 session per week in the remaining 12 weeks. CBT was delivered according to established protocols which comprised behavioral activation and cognitive restructuring. Optional elements such as coping and social skills training, perfectionism, and self-criticism, were also included to address individual maintaining factors. Some treatment sessions were audio-recorded and reviewed by an independent evaluator using the Cognitive Therapy Scale-Revised (CTS-R) to assess therapists’ adherences. Scores met the established threshold of acceptable competence in delivering CBT (CTR-S: 50.43 $\pm$ 4.26). The primary outcome was the Montgomery-Åsberg Depression Rating Scale (MADRS) to assess depression severity. It was administered by the study coordinator and was completed at baseline and every second week during the therapy.

### EEG data recording

EEG data were collected at week 0, week 2, and week 16 of treatment, but only data at baseline (week 0) and week 2 were used in this study. EEG data were recorded using a 64-channel SynAmps 2 EEG system (Neuroscan, Compumedics USA, USA). The International 10–10 system was used for the placement of electrodes. The impedance level of each electrode was lowered to ≤ 5 kΩ, and an electrode positioned posterior to the Cz electrode was used as a reference. Recordings were done at a 10 kHz sampling rate, with a direct current and a low-pass filter. Five minutes of EEG activity were recorded from all participants during eyes-closed resting condition.

### EEG data processing

EEG data were preprocessed using EEGLAB toolbox^84^. Datasets were first standardized to those collected in previous CAN-BIND studies for potential future comparisons. The standardization involved the selection of 58 common channels in all datasets, a re-referencing to average, and a resampling to 512 Hz. A customized, fully automatic pipeline was then used to clean the data. This pipeline was adapted from the ERPEEG toolbox^85^. First, data were high-pass filtered at 0.5 Hz with a second-order Butterworth IIR filter. The EEGLAB plugin clean_rawdata was then used to detect bad channels and bad segments of data. In this step, bad channels were deleted, and bad segments were corrected. Power line artifacts were removed using ZapLine method^86^. Independent component analysis (ICA) was then conducted. Data were temporarily high-pass filtered at 1 Hz for better ICA decomposition^87^, and the ICLabel algorithm^88^ was used to remove components associated with recurring artifacts such as eye movement, eye blinks, muscle, and cardiac artifacts from the original data filtered at 0.5 Hz. Finally, bad channels were interpolated using spherical interpolation, and the data were again re-referenced to average.

### EEG power spectral density

Using Welch’s method, periodograms derived from 2-s non-overlapping windows were averaged to get an estimated power spectral density (absolute power) of each channel from 0.5 to 50 Hz with a frequency resolution of 0.5 Hz. The relative power was obtained by taking the ratio of the absolute power at each frequency and the total sum of absolute power. Internal consistency of relative power measures was also estimated (Supplementary Information).

### EEG source localization

EEG sources were reconstructed using Brainstorm software^89^. For each participant, eyes-closed resting condition EEG data were imported into Brainstorm software^89^. First, the locations of the 58 EEG channels were co-registered to the ICBM152 MRI template. The OpenMEEG BEM head model^90^ was then used to calculate the forward model. An identity matrix was used as noise covariance. The inverse problem was derived from the minimum norm estimate model sLORETA^91^, and the solution space was constrained to the cortex surface. In the MNI coordinate space, the Destrieux Atlas^92^ was used, providing 148 cortical regions that served as regions of interest. Finally, relative power measures were computed at these 148 reconstructed sources for all subjects.

### Statistical analysis

Demographic and clinical data were compared between responder and non-responder groups using independent-samples *t* test or Chi-squared test, wherever appropriate.

Analysis of variance was performed to test the differences in relative power (0.5-50 Hz frequencies) between responders and non-responders at baseline and week 2. The main effect of CBT Response (responder, non-responder) and Time (baseline, week 2), and the interaction effect of CBT Response x Time were evaluated across 58 channels in sensor space and 148 regions of interest in source space. For p*ost-hoc* comparisons, when applicable, independent-sample t-statistics were used across all 58 channels and across frequency bands: delta band (0.5–4 Hz), theta band (4–8 Hz), alpha band (8–12 Hz), beta band (12–30 Hz), and gamma band (30–50 Hz). Cluster-based permutation testing^93^ was applied separately within each frequency band to further correct for multiple comparisons. Initial t-values exceeding an a priori threshold of p < 0.05 were clustered together based on adjacent frequency bins and neighboring electrodes. A minimum of 2 neighboring electrodes was considered for a selected sample to be included in the cluster. All *t* values within every cluster were summed to build cluster-level statistics. Finally, using a Monte Carlo method with 2000 permutations, a distribution of the maximum cluster-level statistics was obtained, and the significance of each cluster in the original data was set at p < 0.025 (single-tailed). In source space, the average value of the relative power was calculated within frequency bands in which a significant cluster was found at the sensor space level. *Post-hoc* independent-sample t-statistics were then conducted across all 148 regions of interest and t-values exceeding a threshold of p < 0.05 were considered significant. As correction for multiple comparisons was conducted at the sensor level, and our hypotheses were only about the existence of group differences with no a priori specific brain area being involved, correction for multiple comparisons was not required at the source level^94^.

Further analyses were performed with subdomains of MADRS score^95^. More details are given in the Supplementary Information and the results can be found in Supplementary Fig S5.

### Predictive analysis

Logistic regression models were used to assess the predictive value of relative power to classify participants’ responses to CBT. Logistic regression models were only assessed for relative power measures that were significantly different between responders and non-responders. In sensor space, an average of the relative power measures in the related significant clusters was calculated to obtain a single value per patient. In source space, a single value was obtained for each frequency band by averaging of the relative power across the regions of interest found significantly different. The level of prediction of these single values was quantified by the receiver operating characteristic (ROC) curve, plotting the sensitivity and the sensibility and across all possible thresholds. The area under the curve (AUC) was calculated to determine the significance of the prediction.

Based on previous studies showing that early improvement in symptoms predicts better treatment outcomes^96–98^, we also examined the predictive value of reduction in MADRS score from baseline to week 2 to classify participants' responses to CBT as an exploratory analysis. The results can be found in Supplementary Figs S6 and S7.

All analyses were performed using MATLAB (Version R2020b; MathWorks, Natick, MA, USA).

### Ethics declaration

The authors assert that all procedures contributing to this work comply with the ethical standards of the relevant national and institutional committees on human experimentation and with the Helsinki Declaration of 1975, as revised in 2008.

## Supplementary Information

Supplementary Information.

## Data Availability

Data can be made available upon reasonable request to the corresponding author. Each request will be processed in consultation with the related research ethics boards and institutional data sharing policies and guidelines.
