# Supplementary material for: Resting-state EEG delta and alpha power predict response to cognitive behavioral therapy in depression: a Canadian biomarker integration network for depression study
Source: Sci Rep. 2023 May 24;13:8418. doi: 10.1038/s41598-023-35179-4 (PMC10209049; doi:10.1038/s41598-023-35179-4)
Supplement: Supplementary file 1 — Supplementary Information. [file 41598_2023_35179_MOESM1_ESM.docx]

***Supplementary Information***

**Resting-state EEG Delta and Alpha Power Predict Response to Cognitive Behavioral Therapy in Depression: A Canadian Biomarker Integration Network for Depression Study**

**Supplementary Methods and Materials**

**Eligibility for CBT**

Study exclusion criteria included: (1) lifetime diagnosis of schizophrenia, schizoaffective disorder, bipolar disorder; (2) current substance use disorder (except tobacco or caffeine); (3) current psychotic symptoms; (4) acute suicide risk; (5) psychological treatment for depression initiated during the past three months; (6) pharmacological treatment for depression initiated/changed during the past three months; (7) previous non-response to two or more adequate trials of pharmacotherapy; (8) current significant neurological disorder, head trauma, or unstable medical conditions.

**Internal Consistency of Relative Power**

We evaluated the split-half internal consistency of relative power at baseline and week 2. EEG data were split in half and relative power was derived for both half segments. Cronbach’s alpha coefficient was then calculated for each frequency (0.5-50Hz) and each electrode. To get an estimate of internal consistency, Cronbach’s alpha coefficient was averaged across frequencies and across the 58 electrodes.

**Analyses with subdomains of MADRS score**

To assess if differences observed in relative power measures (baseline and early changes) were associated with improvements in specific symptoms, we performed the analyses again with 4 subdomains of MADRS score: sadness, neurovegetative, detachment and negative thoughts^1^. Participants with a reduction of 50% or more were compared to participants with a reduction of 50% or less in each subdomain. Similar to the analyses performed between responders and non-responders to CBT, cluster-based permutation testing was applied separately within each frequency band to further correct for multiple comparisons.

**Predictive analysis**

We evaluated the predictive value of early percentage change in MADRS score from baseline to week 2 to classify responders and non-responders to CBT. A secondary analysis was also conducted to classify remitters and non-remitters to CBT based on their post-treatment MADRS score ^2^. A participant was defined as remitter if they achieved a MADRS score of 10 or less at week 16, and as non-remitter if the score was more than 10 ^3^.

**Supplementary Results**

**Relative Power EEG Spectra**

The average relative power EEG spectra was calculated for all participants at baseline and week 2. It was then averaged per groups (responders and non-responders) and across the 58 electrodes (**Fig S1**).

**Internal Consistency of Relative Power**

Cronbach’s alpha coefficient averaged across frequencies and across electrodes was 0.911 at baseline, and 0.925 at week 2, respectively. For visualisation purposes, we plotted the coefficients averaged across frequencies, and averaged across electrodes, separately (**Fig S2**).

**Relative Power at Baseline**

Of the thirty-four participants completed who completed the EEG session at baseline, nineteen were responders, and fifteen were non-responders. Analysis in the sensor space revealed a group difference with responders exhibiting lower delta activity compared to non-responders (negative cluster, p = 0.016, Cohen d = 0.882, **Fig S3**). In the source space, this difference was identified in several brain areas, including bilateral precentral gyrus and sulcus, bilateral central sulcus, left postcentral gyrus, bilateral precuneus, bilateral superior and inferior parietal gyri, left superior temporal sulcus, left middle occipital gyrus, and bilateral posterior cingulate cortex (PCC) (**Fig S3**).

The difference observed between responders and non-responders in the delta band at baseline was found to be predictive of the treatment outcome (AUC = 0.726, p = 0.008 in the sensor space, and AUC = 0.737, p = 0.007 in the source space, **Fig S4**).

**Analyses with Subdomains of MADRS Score**

Number of participants having a reduction of 50% or more differed for each subdomain and are reported in Table S1.

At baseline, the analyses with subdomains of MADRS score did not lead to significant clusters after correction. Early changes were found to be associated with improvements in subdomains. Significant clusters were observed in delta band with participants, who had stronger improvement in sadness and negative thoughts subdomains, exhibiting an early increase in delta activity (positive clusters, p = 0.024, Cohen d = 0.3123, and p = 0.022, Cohen d = 0.3285, respectively, Fig S5). Significant clusters were also observed in beta band with detachment and negative thoughts subdomains (positive clusters, p = 0.023, Cohen d = 0.0633, and p = 0.006, Cohen d = 0.2361, respectively, **Fig S5**).

**Predictive Value of Reduction in MADRS Scores from Baseline to Week 2**

Thirty-six patients had their MADRS scores assessed both at baseline and at week 2. Early reduction in MADRS score from baseline to week 2 was found to not be predictive of treatment responses (AUC = 0.513, p = 0.717, **Fig S6**) or treatment remissions (AUC = 0.574, p = 0. 452, **Fig S7**).

|  |  |  |  | Participants with |  | Participants with |
| --- | --- | --- | --- | --- | --- | --- |
|  |  |  |  | reduction >= 50% |  | reduction < 50% |
| Variable | | |  | n |  | n |
| Total MADRS | |  |  | 16 |  | 14 |
| Subdomain sadness | |  |  | 19 |  | 11 |
| Subdomain neurovegetative | | |  | 16 |  | 14 |
| Subdomain detachment | |  |  | 18 |  | 12 |
| Subdomain negative thoughts | | |  | 19 |  | 11 |

**Table S1. Number of participants in each group for subdomains of MADRS score analyses.**


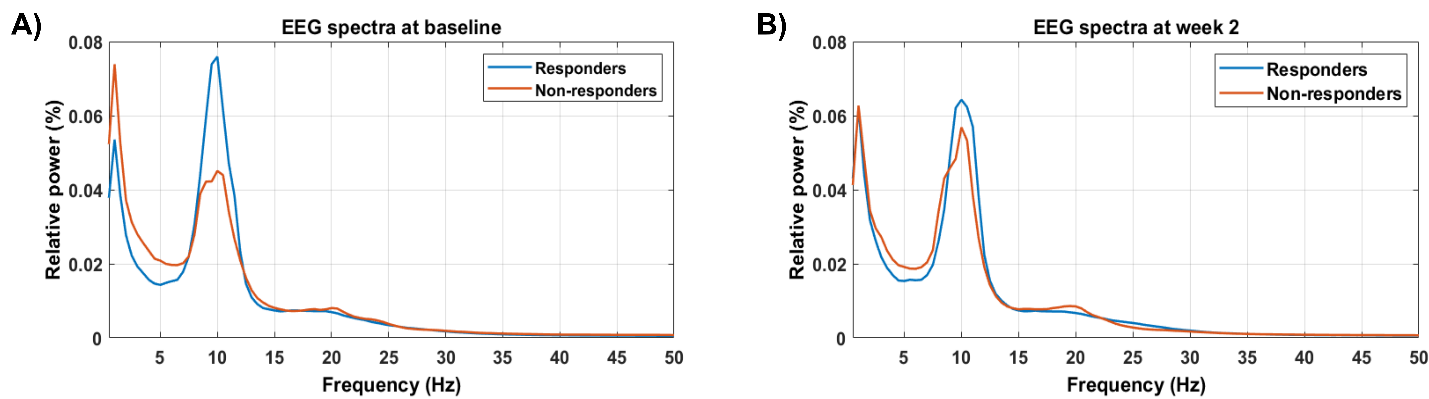


**Supplementary Figure S1. Relative power EEG spectra at baseline and week 2.**

**(A, B)** The x-axis shows frequencies from 0.5 to 50Hz. The y-axis shows the relative power in percentage. Image A shows the average relative EEG spectra for responders (in blue) and non-responders (in red) at baseline. Image B shows the average relative EEG spectra for responders (in blue) and non-responders (in red) at week 2.


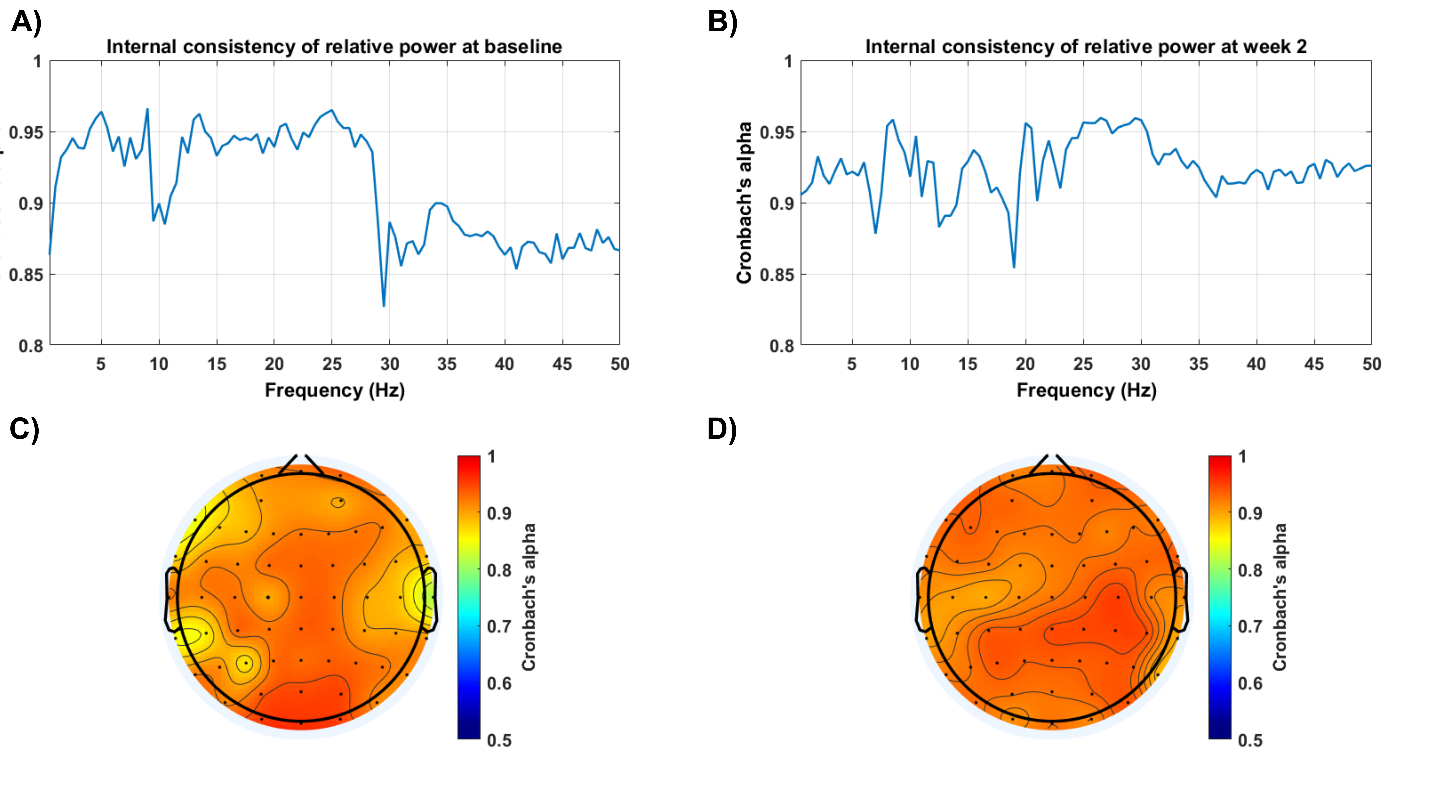


**Supplementary Figure S2. Split-half internal consistency at baseline and week 2.**

**(A, B)** The x-axis shows frequencies from 0.5 to 50Hz. The y-axis shows Cronbach’s alpha coefficient. Internal consistency was averaged across all 58 electrodes at baseline (left) and week 2 (right). **(C, D)** Topographies illustrate Cronbach’s alpha coefficient averaged across frequencies at baseline (left) and week 2 (right).


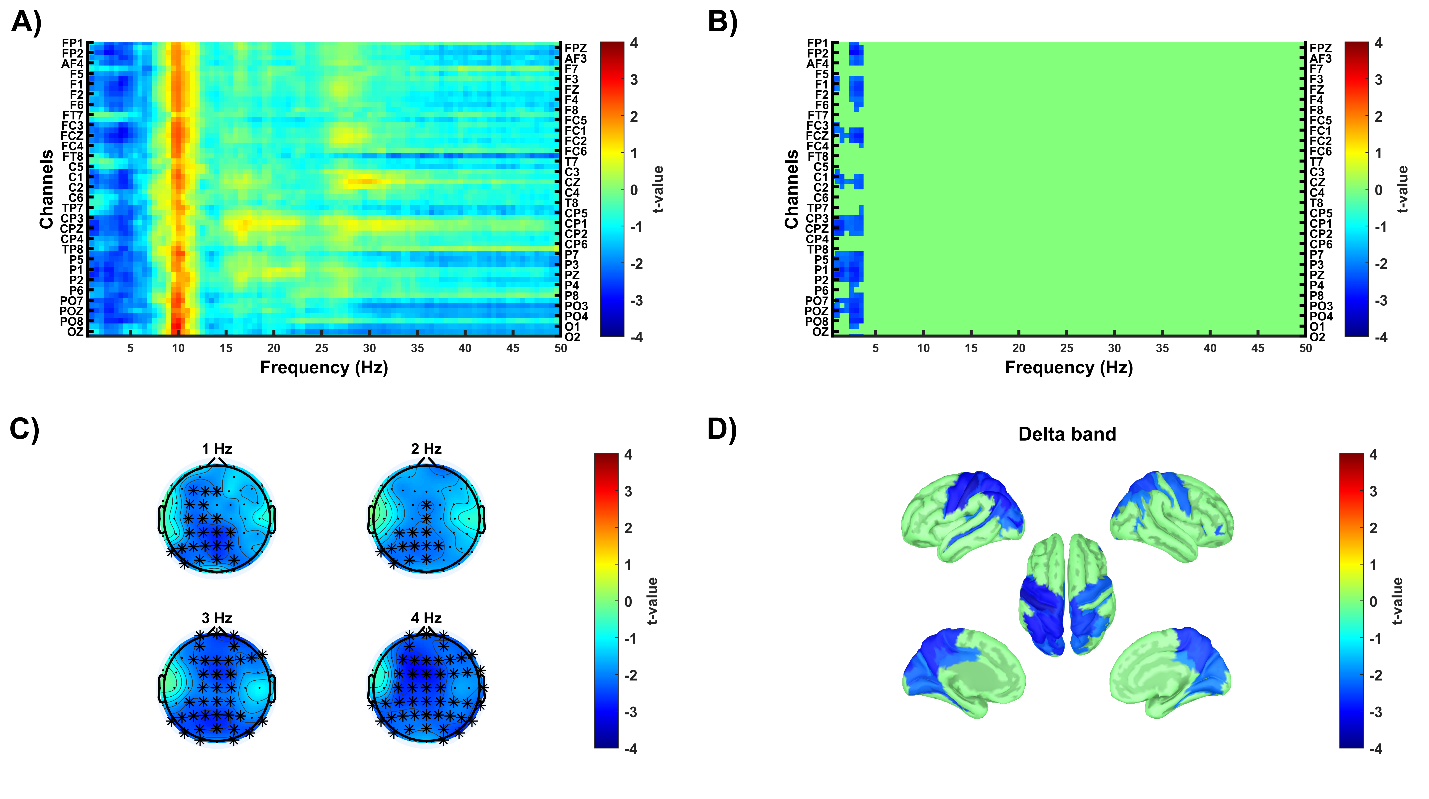


**Supplementary Figure S3. Differences in relative power at baseline between responders and non-responders.**

Cold colors show lower relative power in responders compared to non-responders. Warm colours show higher relative power in responders compared to non-responders. **(A, B)** The x-axis shows frequencies from 0.5 to 50Hz. The y-axis shows all electrodes from 1 to 58. Image A shows uncorrected t-value map, image B shows significant clusters (p < 0.025, single-tailed, cluster corrected for multiple comparisons) **(C)** Topographies illustrate t-values at different frequencies with stars indicating electrodes that belonged to the significant cluster. **(D)** Cortical maps depict source-localized regions (p < 0.05, uncorrected) in the frequency band in which the cluster was found at the sensor space level.


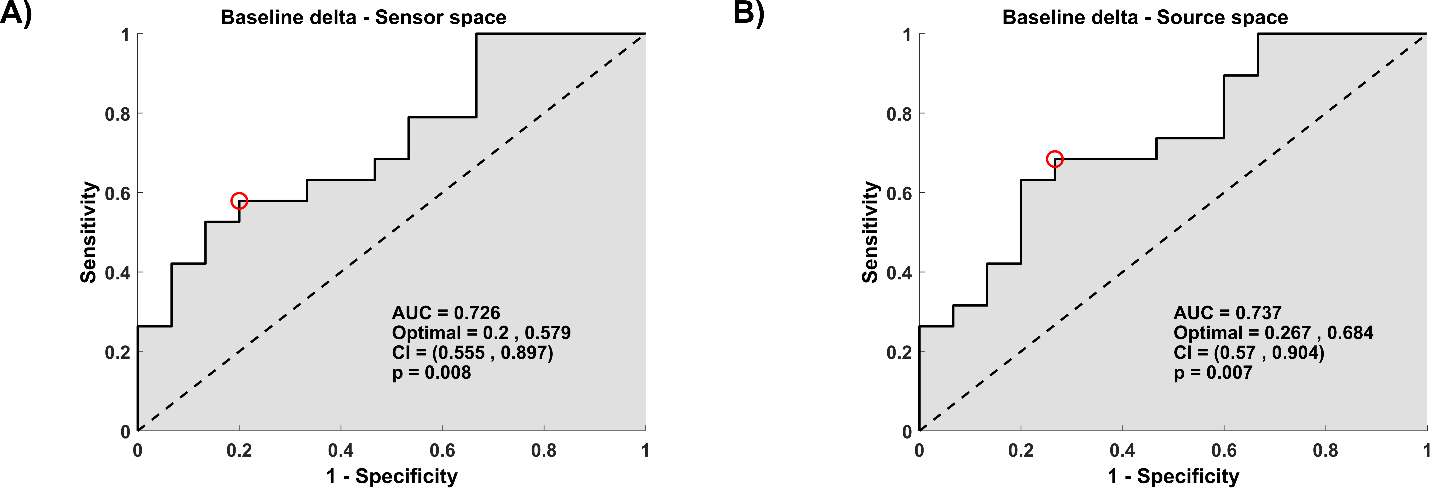


**Supplementary Figure S4. Relative power in the delta band at baseline predicts improvement in depressive symptoms.**

**(A)** The plot depicts the ROC curve across all possible threshold values of the predictor for average power relative value within the cluster found in the delta band at the sensor space **(B)** The plot depicts the ROC curve across all possible threshold values of the predictor for average power relative value in the delta band across regions found significant at the source space. **(A, B)** In both plots, x-axes represent false-positive rates (1-specificity), y-axes the true positive values (sensitivity). Here, specificity corresponds to percentage of non-responders who were predicted to be non-responders, and sensitivity corresponds to percentage of responders who were predicted to be responders. The red circle shows the optimum operating point of the ROC curve.


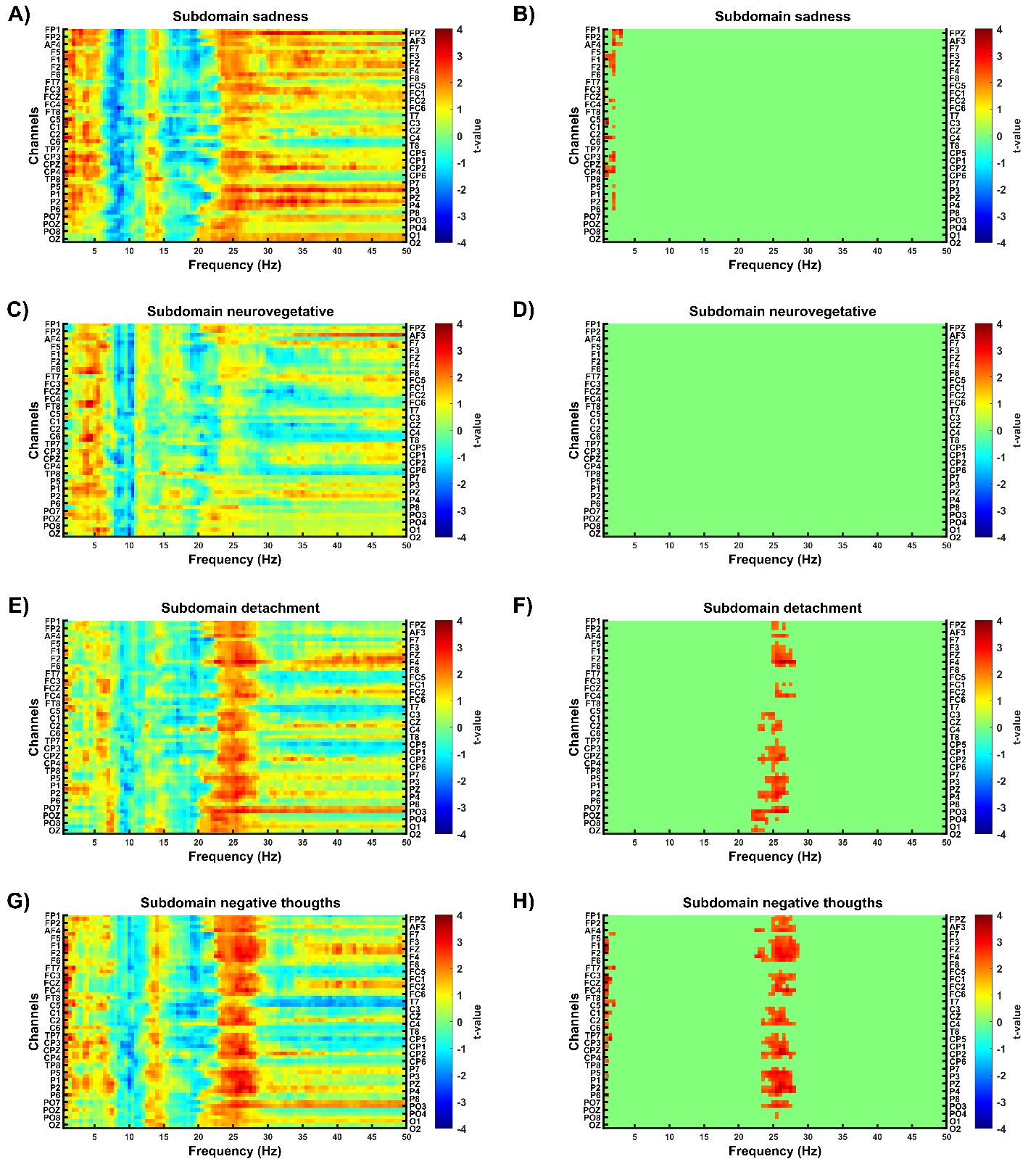


**Supplementary Figure S5. Differences in relative power early changes for each subdomain of MADRS.**

Cold colors show lower relative power changes in participants with improvement greater than 50% compared to participants with improvement lower than 50% in each subdomain of MADRS: sadness (A, B), neurovegetative (C, D), detachment (E, F), and negative thoughts (G, H). Warm colours show higher relative power changes in participants with improvement greater than 50% compared to participants with improvement lower than 50% in each subdomain of MADRS. **(A, B, C, D, E, F, G, H)** The x-axis shows frequencies from 0.5 to 50Hz. The y-axis shows all electrodes from 1 to 58. Images A, C, E and G show uncorrected t-value map, images B, D, F and H show significant clusters (p < 0.025, single-tailed, cluster corrected for multiple comparisons).


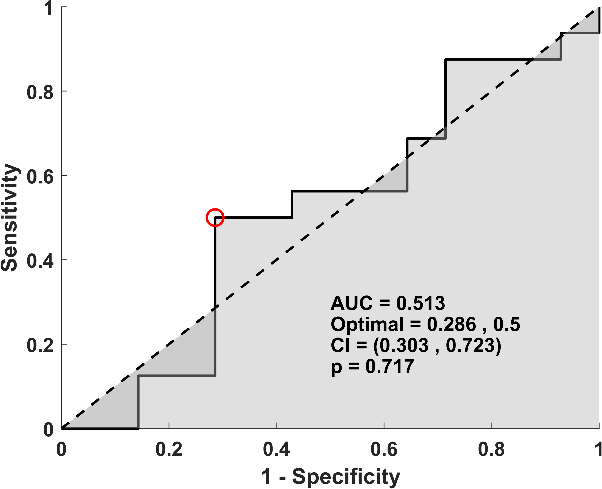


**Supplementary Figure S6. Early reduction in MADRS score from baseline to week 2 does not predict treatment responses.**

The plot depicts the ROC curve across all possible threshold values of the predictor for reduction in MADRS scores from baseline to week 2. X-axe represents false-positive rates (1-specificity), y-axe the true positive values (sensitivity). Here, specificity corresponds to percentage of non-responders who were predicted to be non-responders, and sensitivity corresponds to percentage of responders who were predicted to be responders. The red circle shows the optimum operating point of the ROC curve.


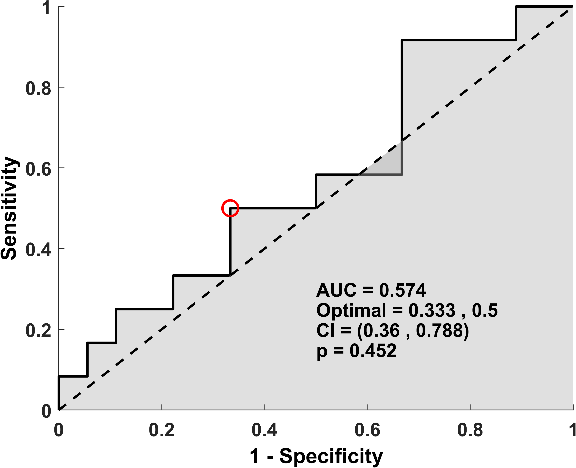


**Supplementary Figure S7. Reduction in MADRS scores from baseline to week 2 does not predict treatment remissions.**

The plot depicts the ROC curve across all possible threshold values of the predictor for reduction in MADRS scores from baseline to week 2. X-axe represents false-positive rates (1-specificity), y-axe the true positive values (sensitivity). Here, specificity corresponds to percentage of non-remitters who were predicted to be non-remitters, and sensitivity corresponds to percentage of remitters who were predicted to be remitters. The red circle shows the optimum operating point of the ROC curve.

**Supplementary References**

1. Quilty, L. C. *et al.* The structure of the Montgomery–Åsbergdepression rating scale over the courseof treatment for depression. *Int. J. Methods Psychiatr. Res.* **22**, 175–184 (2013).

2. Vickers, A. J. The use of percentage change from baseline as an outcome in a controlled trial is statistically inefficient: A simulation study. *BMC Med. Res. Methodol.* **1**, 1–4 (2001).

3. Hawley, C. J., Gale, T. M. & Sivakumaran, T. Defining remission by cut off score on the MADRS: selecting the optimal value. *J. Affect. Disord.* **72**, 177–184 (2002).
